# Open complete anterior dislocation of the sacro-iliac joint in a 4-year-old boy: a case report of a rare injury with 5-year follow-up

**DOI:** 10.1007/s11751-017-0294-6

**Published:** 2017-09-09

**Authors:** Walid A. Elnahal, Mahmoud Fahmy, Mehool Acharya

**Affiliations:** 1 Trauma and Orthopaedics Department, Faculty of Medicine, Cairo University, Giza, Egypt; 2 Trauma and Orthopaedics Department, North Bristol NHS Trust, Bristol, UK

**Keywords:** Pelvic fracture, Pediatric pelvic fracture, Pediatric trauma

## Abstract

Pelvic fractures are uncommon in children and account for between 0.3 and 7.5% of all pediatric injuries. Open pelvic fractures only account for up to 12.9% of all pediatric pelvic fractures. An unusual case of open complete anterior sacro-iliac joint dislocation in a 4-year-old boy is presented with a long-term follow-up. The multidisciplinary approach is reported with review of the current literature. A 4-year-old male presented to our institution in January 2012 after having been run over by a tractor. He presented with gross hemodynamical instability, MISS of 25, and an unstable lateral compression type III pelvic fracture with complete anterior dislocation of the left hemipelvis and a groin wound extending into the left thigh. The patient was managed in accordance with the ATLS and open fracture guidelines. Reduction in the dislocated SI joint was achieved via a posterior approach to the SI joint, followed by fixation with 2K wires in S1 and S2 sacral segments, with an anterior external fixator. Pelvic asymmetry post-reduction was 0.9 cm, compared to 16 cm post-injury, and asymmetry persisted till final follow-up at 5 years. At 5 years, patient regained full function, including recreational sport activities. Patients scored a 96/96 on the Majeed score (after excluding 4 points for sexual function). We believe that posterior reduction in an anteriorly dislocated SI joint in the pediatric population is a viable option. A coordinated, multidisciplinary approach and restoration of pelvic ring stability can lead to optimal outcome.

## Introduction

Pelvic fractures are an uncommon injury and account for between 0.3 and 7.5% of all pediatric injuries. Open pelvic fractures are even less common and only account for between 0.6 and 12.9% of all pelvic fractures in children. The majority of these injuries are caused by road traffic accidents [4, 7, 8].

The differences in physiology and anatomy account for the difficulty in managing these fractures. Pediatric patients have different responses when compared to adult patients with regard to their adaptive mechanisms following trauma. Differences in heart rate, blood pressure, response to blood loss are challenging during the initial resuscitation phase [15].

Additionally, an apparently benign pelvic injury may conceal a much more serious blunt abdominal or soft tissue injury due to the relative resilience of the bony structure in children. Furthermore, injury to the growth centers may alter the long-term outcomes of these injuries [3].

The modified Trode and Zieg classification is commonly used for pediatric fracture injuries, where type IIIB injuries involve the anterior and posterior ring and are associated with a higher incidence of associated injuries, need for transfusion and ICU admission. Type IV is multifocal injuries that represent the highest spectrum of instability [13]. The Young and Burgess classification is also used to describe pediatric pelvis fractures based on the mechanism of injury, and the Tile classification which is based on the stability of the pelvic ring.

Hemodynamically unstable patients with unstable anterior and posterior pelvic ring injuries are usually managed acutely. Management consists of pelvic binder application, external fixation, pelvic packing or angiography [3].

We present an unusual case of pelvic injury in a 4-year-old boy with complete anterior dislocation of the sacro-iliac (SI) joint that presented acutely with hemodynamical instability to the emergency department in Cairo University Hospitals, together with a groin laceration following a runover tractor injury.

An informed consent and approval of publication were obtained from the patient’s first degree relative prior to submission.

## Patients and methods

The 4-year-old boy was runover by a tractor and presented to our trauma center in January 2012 at approximately 3 am. On presentation, he had a GCS of 12 with gross hemodynamic instability. Initially, he was managed using the ATLS guidelines. Resuscitation was carried out with red blood cells and plasma, with a ratio of 2:1. Secondary survey revealed pelvic instability, external rotation deformity of the left lower limb, a groin wound extending to the left thigh overlying the course of the major vessels with gross contamination, intact bilateral dorsalis pedis pulses and posterior tibial pulses.

**Fig. 1:**
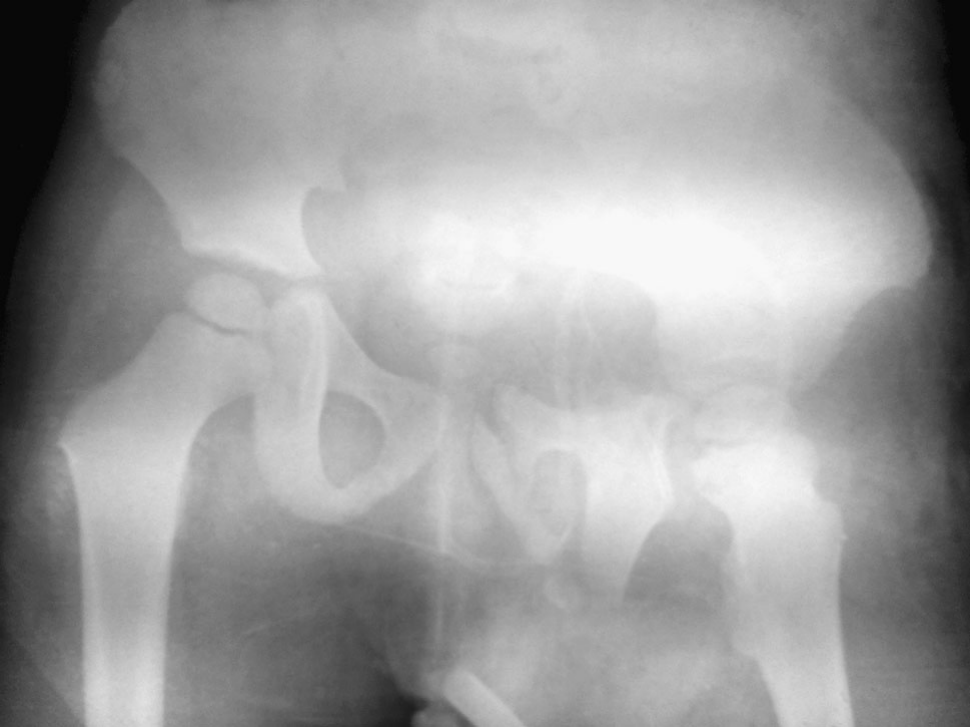
Post-injury AP radiograph showing external rotation deformity of the left hemipelvis and pelvis asymmetry was measured at 16 cm

**Fig. 2:**
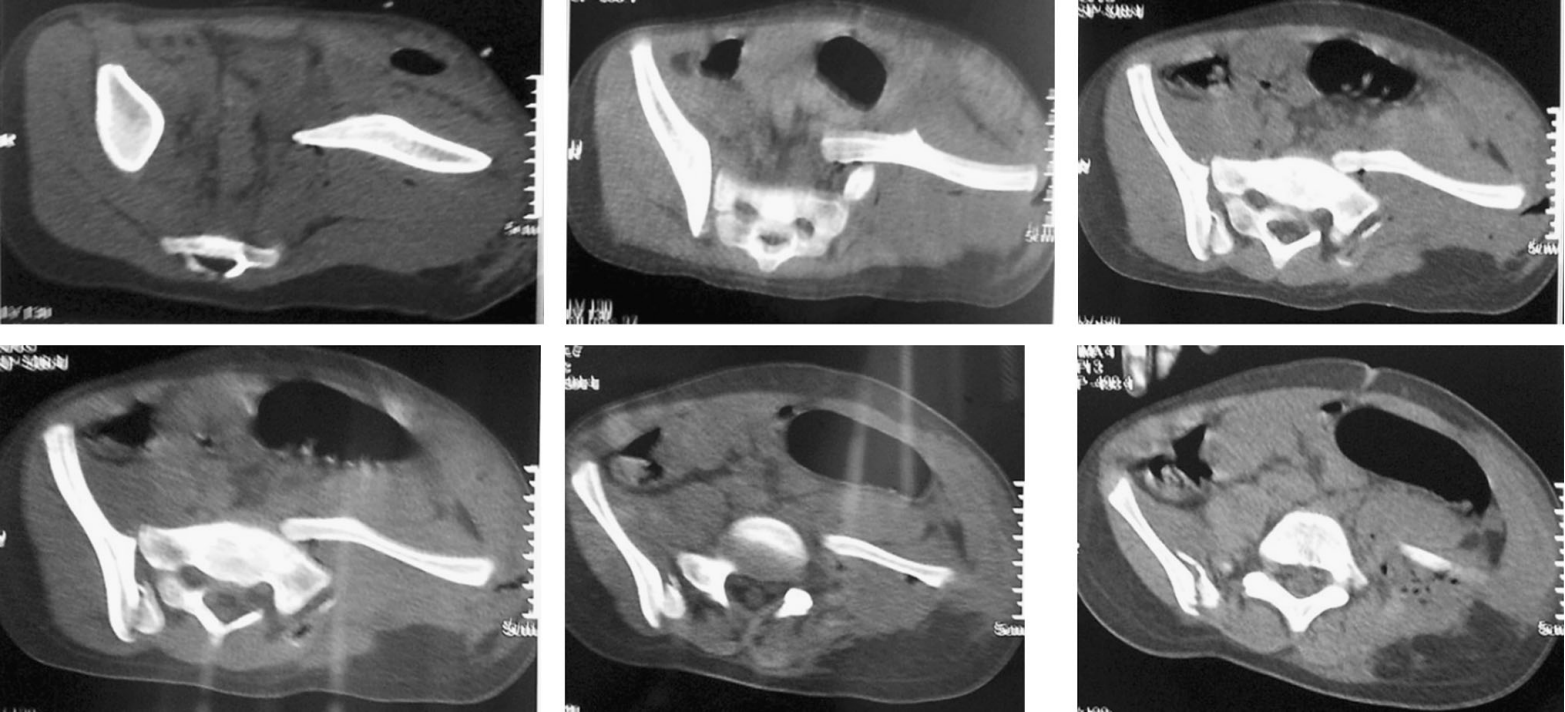
Axial cuts post-injury showing a right crescent fracture with a left external rotational injury

The initial trauma computed tomography (CT) scan of the head, chest and abdomen excluded injuries to these body compartments. The pelvic injury was classified as a lateral compression type III injury, with a crescent fracture (fracture dislocation of the sacro-iliac joint) of the right hemipelvis and external rotation deformity of his left hemipelvis, with complete anterior dislocation of the left ilium over the sacrum. The fracture was also classified according to the Trode and Zieg classification and as type IV (Figs. 1, 2). Modified Injury Severity Score (MISS) was calculated at 25 (critical pelvic injury and moderate thigh injury) [11].

There was a wound that measured 12 cm located over the left groin extending down to the left thigh.

The wound was managed according to our local open fracture protocol. The young boy was given intravenous flucloxacillin in the emergency department. All foreign material and any gross contamination were removed, and moist dressing was applied to the wound.

Application of temporary pelvic binder was not feasible due to the pattern of pelvic injury and the fear that the binder would make the displacement worse.

The young patient was taken to theaters in the early hours of the morning (4 h post-presentation), for wound exploration, debridement and reduction in the pelvic ring.

Wound exploration revealed no injury to the major neurovascular structures, and the wound did not extend beyond the abdominal muscles or into the hip joint. The wound was debrided and closed primarily.

The patient was placed in the prone position, and through a posterior approach to the SI joint, the dislocation was identified and reduced by gently levering the ilium over the SI joint with a curved retractor.

The pelvis was secured posteriorly with 2 × 2.7 mm Kirschner wires through the S1 and S2 sacral segments, and the use of 4 mm percutaneous screws was not used due to the relatively small passage available in the sacrum.

**Fig. 3:**
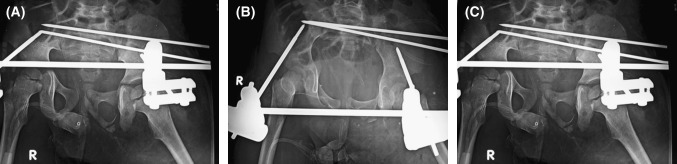
Post-op X-rays **a** AP view, **b** inlet view, **c** outlet view, fixation with 2 × 2.7 cm Kirchner wires and anterior external fixator. Pelvic asymmetry was measured as 0.9 cm

Anteriorly, the patient pelvic ring was stabilized with an external fixator (Fig. 3).

**Fig. 4:**
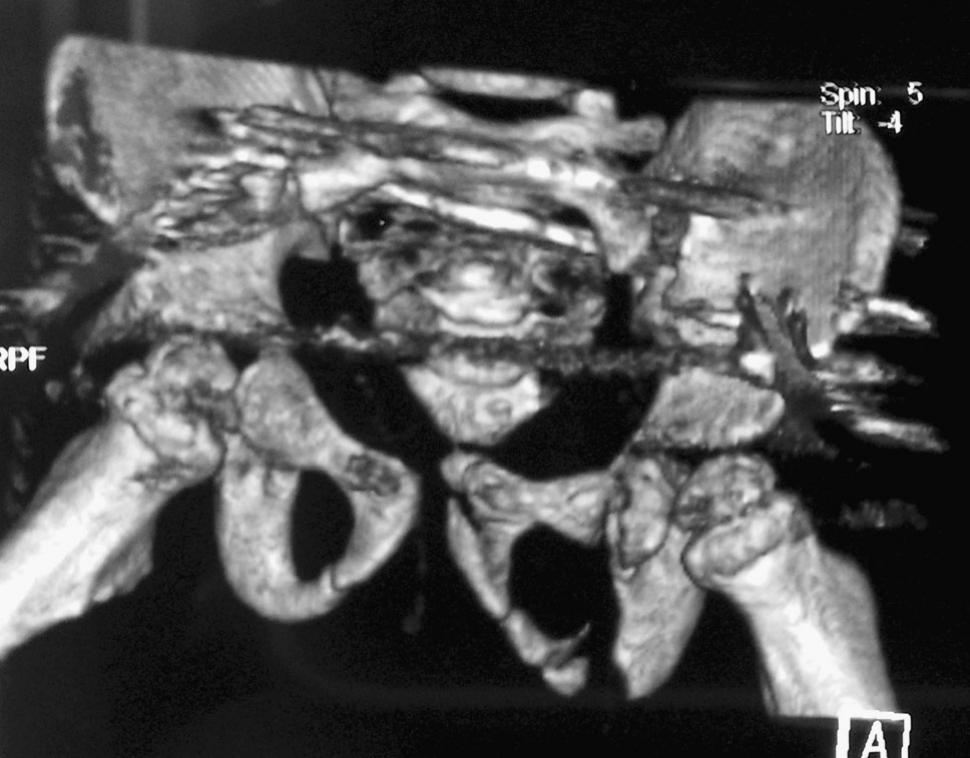
Post-op CT, showing some residual external rotational deformity in the left hemipelvis, the wires in S1 and S2, protruding into the soft tissue (in–out–in position)

The patient was then admitted to HDU for further resuscitation and monitoring. Post-reduction CT angiography revealed intact bilateral iliac arteries, and postoperative pelvic CT scan revealed that the wires were partially protruding into the true pelvis, but clinically the patient was neurovascularly intact, and decision was made to leave them till fracture union (Fig. 4).

Clinically, there was no leg length discrepancy or rotational deformity. Patient was discharged 14 days post-injury and followed up 2 weeks later for wound check. The external fixator and wires were removed at 6 weeks, and patient was put into a spica cast for a further 4 weeks.

The patient was seen at 6-month intervals for one year and then returned annually for the following 4 years. Radiological assessment with X-rays and functional assessment using a modified version of the Majeed score (where the best possible score is 96, due to inability to assess the sexual function) were used at each follow-up visit [10].

Pelvic asymmetry was assessed on the AP radiographs prior to reduction, and with each follow-up X-ray, using the method described by Smith et al. [14].

## Results

Pelvic asymmetry following initial injury was measured as 16 cm, compared to 0.9 cm pelvic in the immediate postoperative radiographs.

The groin wound healed without any complications and the patient started weight bearing after removal of the spica cast at 10 weeks post-injury. At one year post-injury, the patient returned to normal activities including normal attendance at school and participation in all school activities. At one year, the modified Majeed score was 89/96 and this reached 96/96 at final follow-up. At 5 years post-injury, the patient was able to participate in regular recreational sporting activities including football/

**Fig. 5:**
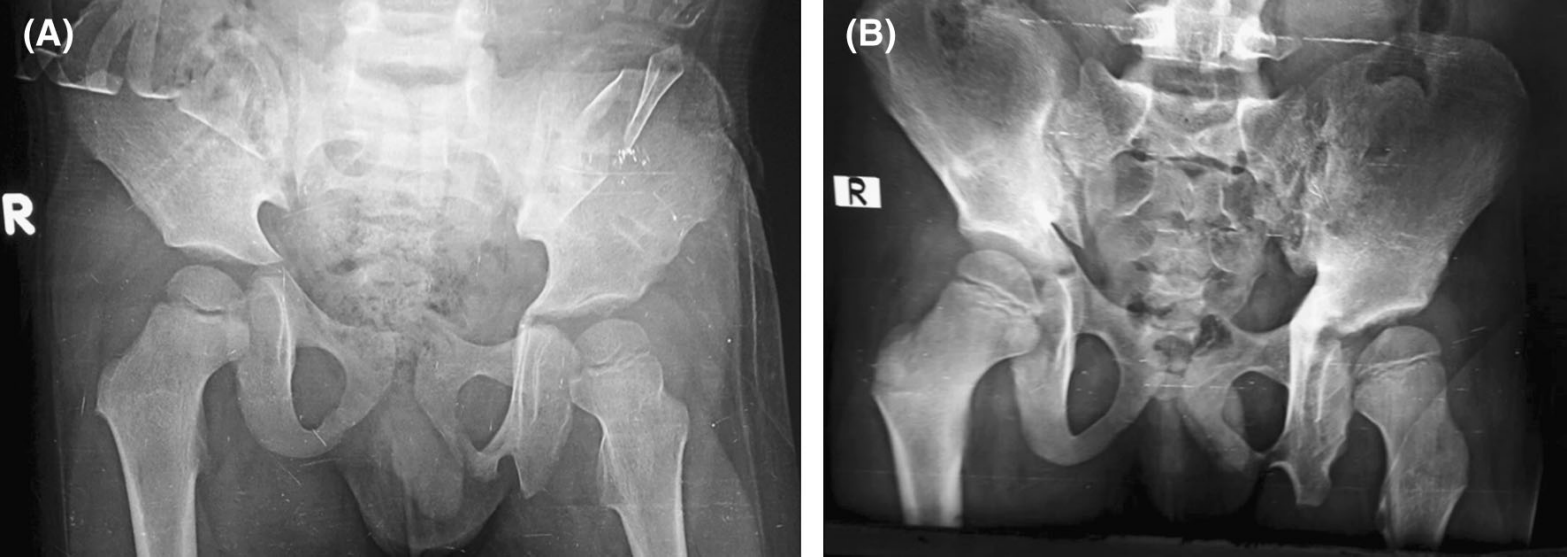
**a** One-year follow-up AP films. **b** 5-year follow-up AP films. Both films show persistent pelvic asymmetry at approximately 0.9 cm, some sclerosis around the triradiate cartilage at 5 years

At final follow-up, X-rays showed some residual external rotational deformity with a pelvic asymmetry of 0.9 cm, a fused triradiate cartilage on the left side and dysplastic changes of the left hip (Fig. 5).

## Discussion

We present a rare and challenging case of a 4-year-old boy who sustained an open complete anterior dislocation of the ilium. After initial resuscitation, removal of gross contamination from the wound, and detection of the dorsalis pedis pulse, the consensus was to proceed to theater at the earliest possible opportunity.

The dislocation was reduced via a posterior approach to the SI joint. The rationale behind this was to avoid the iliac neurovascular bundle and to avoid disrupting the pelvic hematoma and the tamponade effect.

The surgeons were cautious regarding the use of 4-mm screws to fix the pelvis given the small corridor in the sacrum. Perhaps in an older patient, internal fixation with screws may be possible. However, to date there is no clear consensus in the literature regarding the appropriate age where a S1 screw and S2 screw might be feasible.

Burn et al. [1] reviewed the CT scans of 174 children aged between 2 and 16 years and identified a safe pathway in 4-year-old patients, with a mean pathway width of 8.2 mm (±1.4) for trans-sacral, trans-iliac S1 screws, i.e., screws passing from the posterior ilium, to the sacrum and then to the contralateral ilium. They also identified a pathway width of a 7.2 mm (±0.7) mm at S2. Additionally, at 4 years of age almost 100% of patients had a safe passage for a 6.5-mm SI screw; approximately 70% had a safe passage for trans-sacral–trans-iliac S1 screws, and less than 40% has a safe passage for an S2 screw.

Guimarães et al. [6] reported the use of 7 SI screws (6 were 4-mm-diameter screw, and one 7 mm in an older child), the mean age of their cohort was 8.8 years, range (2–13 years). They used a SI screw in a 2-year-old child, but the SI screw did not pass beyond the level of the sacral foramens, and only fixed the ilium to the sacral alar.

Smith et al. [14] reported on 18 pediatric patients (mean age 6.5 years), which were treated operatively with either anterior external fixators alone (7 patients), or anterior external fixators plus posterior SI screws (11 patients). They found that the asymmetry between the both groups did not differ immediately postoperative, but there was significant asymmetry at the time of union and final follow-up in favor of the group treated with posterior SI screws.

The findings by Smith et al. [14] suggested that in contrast to the previous belief that pelvic fractures in children would eventually remodel; pelvic asymmetry either persisted or increased with time. Currently, there is overall consensus in the literature that unstable pelvic fractures in children require operative intervention [1–3, 5, 9].

This mechanism of injury is not uncommon, but the fact that a tractor was involved contributed to the severity of the injury. The majority of pelvic fracture in children occur due to pedestrian motor vehicle accidents (MVA), followed by passenger MVA; other reported modes of injury include falls, sport injuries, bicycle injuries and driveway injuries [2, 3, 7]. Additionally, Shaath et al. [12] found that children with open triradiate cartilage are more likely to suffer from injuries due to pedestrian MVA, which results in lateral compression-type injuries, while children with open triradiate cartilage are more likely to suffer from passenger MVA, which mimics antero-posterior compression-type injuries.

The described injury represents a severe form of a lateral compression-type injury, which does not precisely fit into the Young and Burgess, Tile or Trode and Zieg classification systems.

The patient returned to normal functional levels at one year, with only mild occasional pain that resolved at 2 years, and remained symptom free till his 5-year follow-up. The long-term outcome is still difficult to predict.

Chia et al. [2] reported a 30% of long-term sequelae following pediatric pelvic fractures, including learning problems, neurological injuries and leg length discrepancies. Mortality is thought to be associated with MISS ≥ 26 and depressed GCS on presentation [2], while poor functional outcomes are correlated with asymmetry at time of injury, and asymmetry more than one cm at time of follow-up [14].

## Conclusion

We believe that any severe pelvic fracture in a child needs to be treated according to ATLS guidelines to rule other life-threatening injuries that might require emergency treatment and surgery. Open pelvic fractures require a coordinated approach to treatment with early administration of antibiotic prophylaxis and immediate management of the wound and other potential visceral injuries. Posterior reduction in an open complete anterior dislocation of the SI joint in the pediatric population can provide a viable option to restore pelvic ring anatomy and stability to achieve optimum outcome.
